# Harnessing glycolysis in gastric cancer: molecular targets, therapeutic strategies, and clinical horizons

**DOI:** 10.3389/fimmu.2025.1628937

**Published:** 2025-09-04

**Authors:** Zexing Shan, Yefu Liu

**Affiliations:** ^1^ Department of Gastric Surgery, Liaoning Cancer Hospital and Institute, Cancer Hospital of China Medical University, Shenyang, China; ^2^ Department of Hepatobiliary and Pancreatic Surgery, Liaoning Cancer Hospital & Institute, Shenyang, Liaoning, China

**Keywords:** gastric cancer, glycolytic metabolic reprogramming, lactate, tumor microenvironment, therapeutic targeting

## Abstract

Gastric cancer (GC) continues to rank among the leading causes of cancer-related mortality globally, with treatment resistance and recurrence posing significant clinical hurdles. While surgical interventions, chemotherapy, and targeted therapies are available, their efficacy in managing advanced or metastatic forms of the disease remains constrained. This review provided an overview of the role of glycolytic reprogramming in gastric cancer, emphasizing the complex regulation by epigenetic mechanisms, non-coding RNAs, post-translational modifications, and oncogenic signaling pathways. This review discusses how epigenetic mechanisms, including m6A methylation and ceRNA networks involving circRNAs and microRNAs, modulate key glycolytic enzymes such as PKM2, HK2, and PGK1, thereby promoting tumor growth, metastasis, and chemoresistance. The study also emphasizes the impact of post-translational modifications like succinylation and ubiquitination on enzyme activity, affecting glycolytic flux and tumor adaptability. Additionally, the article details the crosstalk between glycolytic pathways and oncogenic signaling networks, including hypoxia-inducible factors and YAP/TAZ transcriptional regulators, which sustain tumor stemness and immune evasion. Therapeutic strategies targeting these metabolic vulnerabilities—such as inhibiting m6A regulators, disrupting ceRNA interactions, and modulating enzyme modifications—are discussed as potential approaches to improve gastric cancer treatment. Overall, we underscores the complexity of metabolic regulation in gastric cancer and proposes that targeting its epigenetic and signaling networks offers promising avenues for innovative therapies to overcome resistance and hinder tumor progression.

## 1 Introduction

Gastric cancer (GC) maintains its global dominance as the fifth most prevalent malignancy and third leading contributor to cancer mortality, with disproportionately high disease burden observed worldwide (1–7). While epidemiological trends show declining incidence in some geographical regions, delayed diagnosis persists due to nonspecific early clinical manifestations and inadequate screening biomarkers, culminating in a sobering 5-year survival rate below 30% for advanced-stage patients (8). The molecular pathogenesis of gastric cancer (GC) unfolds through Correa’s multi-step carcinogenic sequence. In this progression, Helicobacter pylori infection acts in synergy with chronic inflammatory processes and the accumulation of genetic/epigenetic aberrations, collectively driving the malignant transformation from gastritis to adenocarcinoma (9–12). Current therapeutic modalities, encompassing surgical resection, cytotoxic chemotherapy, and Human epidermal growth factor receptor 2 (HER2)-targeted agents, demonstrate limited effectiveness against tumor heterogeneity, metastatic dissemination, and therapy resistance mechanisms potentiated by the immunosuppressive tumor microenvironment (TME) (13–16). Even breakthrough immunotherapies exhibit modest clinical responses in GC (17–19), emphasizing the critical imperative to discover innovative therapeutic strategies targeting fundamental biological vulnerabilities such as metabolic reprogramming (20–22).

Metabolic reprogramming represents an essential adaptive mechanism enabling malignant cells to sustain uncontrolled proliferation under nutrient-constrained conditions (23–25). The Warburg effect-characterized by preferential glucose utilization through aerobic glycolysis despite oxygen availability – serves as a cornerstone of this metabolic rewiring in cancer biology (26–29). This bioenergetic shift facilitates rapid ATP generation while accumulating glycolytic intermediates for macromolecule biosynthesis, concurrently establishing an acidic, lactate-enriched TME that fosters immune escape, neoangiogenesis, and metastatic competence (30–35). Molecular orchestrators of this process include hypoxia-inducible factor-1α (HIF-1α), oncogenic kinase cascades, and rate-limiting glycolytic enzymes such as hexokinase 2 (HK2) and lactate dehydrogenase A (LDHA), all frequently overexpressed in malignant lesions (36–41). In GC pathogenesis, Hpylori-induced inflammatory signaling synergizes with oncogenic drivers to amplify glycolytic flux, establishing a self-reinforcing cycle that accelerates tumor progression and therapeutic resistance (42, 43). Preclinical investigations employing glycolytic pathway inhibitors-targeting glucose transporters (GLUTs), LDHA enzymatic activity, or lactate efflux mechanisms-have achieved significant suppression of tumor growth and chemotherapy desensitization, underscoring glycolysis inhibition as a promising therapeutic strategy (44–49).

The glycolytic phenotype exerts multifaceted impacts on gastric carcinogenesis and treatment responses. Gastric cancer (GC) cells display a striking reliance on glycolysis, driven by constitutive activation of the PI3K/AKT/mTOR signaling pathway and stabilization of HIF-1α—effects often amplified by Helicobacter pylori-associated chronic inflammation (50–56). This metabolic adaptation not only fuels unchecked proliferation but also generates an immunosuppressive, pro-metastatic niche through extracellular acidification and lactate accumulation (57–59). Clinically relevant glycolytic markers including HK2 and LDHA demonstrate strong correlations with advanced tumor stage, chemotherapy failure, and poor prognosis. Mechanistically, LDHA-generated lactate enhances β-catenin pathway activation, promoting cancer stem cell maintenance (60, 61). Emerging evidence reveals metabolic heterogeneity across GC molecular subtypes, presenting both challenges and opportunities for precision targeting. While preclinical models demonstrate encouraging antitumor effects with glycolytic inhibitors, clinical translation remains hampered by limited GC-specific trials and incomplete understanding of lactate’s dual metabolic/signaling roles. This comprehensive review analyzes the pathophysiological significance of glycolytic remodeling in GC and evaluates innovative therapeutic approaches, including metabolic inhibitor-immunotherapy combinations and nanoparticle-mediated drug delivery systems, that may overcome current limitations in targeted therapy development.

## 2 Glycolytic reprogramming in malignant progression

### 2.1 Core biochemistry of glycolytic flux

The glycolytic pathway represents an evolutionarily conserved mechanism for cytosolic glucose catabolism, producing both ATP and metabolic precursors critical to cellular homeostasis. Initiated by glucose uptake mediated by the GLUT family of transporters, this ten-step enzymatic cascade includes three irreversible phosphorylation reactions, catalyzed by hexokinase (HK), phosphofructokinase-1 (PFK-1), and pyruvate kinase (PK), respectively (44, 62–66). Following GLUT-mediated cellular entry, glucose undergoes HK-dependent phosphorylation to glucose-6-phosphate (G6P), committing the molecule to glycolytic processing. Subsequent isomerization yields fructose-6-phosphate (F6P), which undergoes PFK-1-catalyzed conversion to fructose-1,6-bisphosphate (F1,6BP) – the pathway’s primary regulatory node through allosteric control by ATP, citrate, and fructose-2,6-bisphosphate (F2,6BP). Cleavage of F1,6BP generates two triose phosphates, with glyceraldehyde-3-phosphate (GA3P) entering the energy-yielding phase through oxidation to 1,3-bisphosphoglycerate, coupled with NADH production. Final steps yield pyruvate, which under normoxic conditions enters mitochondria for oxidative phosphorylation (OXPHOS), while hypoxia prompts lactate dehydrogenase (LDH)-mediated reduction to lactate with concomitant NAD^+^ regeneration – a critical adaptation for glycolytic continuity.

### 2.2 The Warburg effect: metabolic hallmark of malignancy

**Figure 1:**
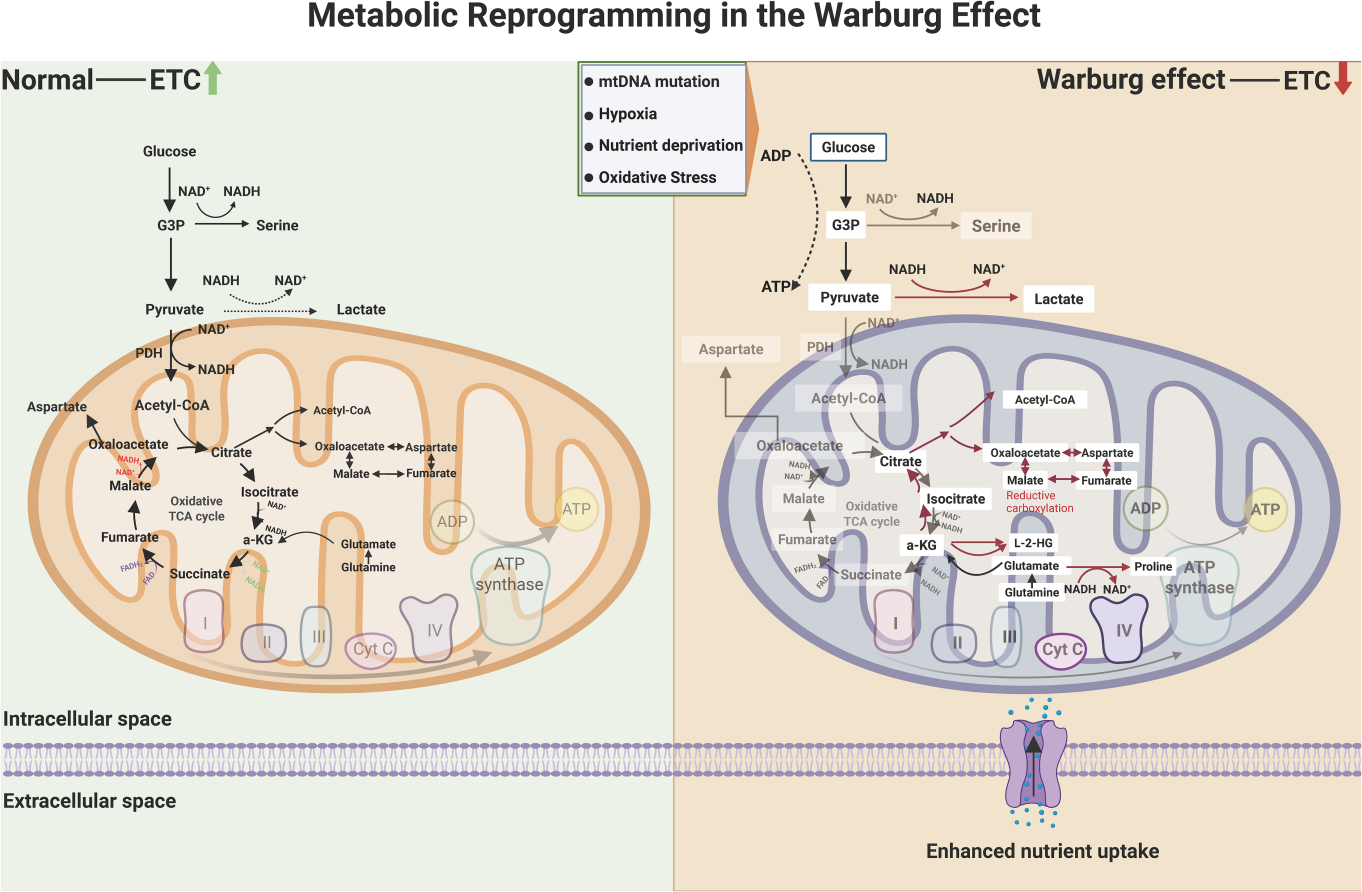
Metabolic circuitry of the Warburg effect in oncogenic adaptation. While normal cells predominantly harness mitochondrial oxidative phosphorylation (OXPHOS) for energy production—coupling the tricarboxylic acid (TCA) cycle with electron transport chain (ETC) activity—cancer cells exhibit a hallmark metabolic divergence. Malignant phenotypes prioritize glycolysis and lactate fermentation, even under oxygen-replete conditions, to sustain biosynthetic demands. This reprogramming arises from mitochondrial insufficiency (e.g., mtDNA lesions, ETC dysfunction) and microenvironmental stressors (hypoxia, nutrient scarcity), which suppress NAD^+^/NADH recycling and amplify reductive glutamine metabolism. The resultant accumulation of oncometabolites, including L-2-hydroxyglutarate (L-2-HG) via α-ketoglutarate (α-KG) reduction, inhibits α-KG-dependent dioxygenases (e.g., TET enzymes, histone demethylases), thereby stabilizing hypoxia-inducible factor 1α (HIF-1α) and silencing tumor suppressors. Concurrently, reductive carboxylation converts α-KG to citrate via isocitrate dehydrogenase (IDH) isoforms, bypassing canonical TCA flux to fuel lipogenesis and macromolecular synthesis. These adaptations collectively drive stemness, immune evasion, and therapeutic resistance through epigenetic dysregulation and redox balance rewiring. (Created by biorender.com **)**.

The Warburg effect epitomizes cancer’s metabolic paradox: preferential reliance on aerobic glycolysis over mitochondrial OXPHOS despite oxygen availability (32, 67, 68). Unlike normal cells that maximize ATP yield via OXPHOS, malignant cells sacrifice energy efficiency to prioritize rapid biomass synthesis and microenvironment remodeling (69–71). This metabolic rewiring arises from mitochondrial dysfunction, impaired electron transport chain (ETC) activity, and microenvironmental stressors including hypoxia and nutrient competition (72, 73). Consequent NAD^+^/NADH ratio reduction triggers two critical adaptations (74–76). L-2-hydroxyglutarate (L-2-HG) accumulates through NADH-dependent reduction of α-ketoglutarate (α-KG), competitively inhibiting α-KG-dependent dioxygenases to disrupt epigenetic regulation and hypoxic signaling. Concurrently, reductive carboxylation of α-KG to citrate sustains lipogenesis under mitochondrial dysfunction. The resultant lactate overproduction creates an acidic extracellular microenvironment, which activates proteolytic enzymes, stabilizes HIF-1α, and induces vasodilation—collectively driving invasion, angiogenesis, and immune evasion. These interconnected processes constitute the metabolic circuitry of the Warburg effect in oncogenic adaptation, as illustrated in **Figure 1**.

### 2.3 Glycolytic enzymes as multifaceted oncogenic drivers

**Figure 2:**
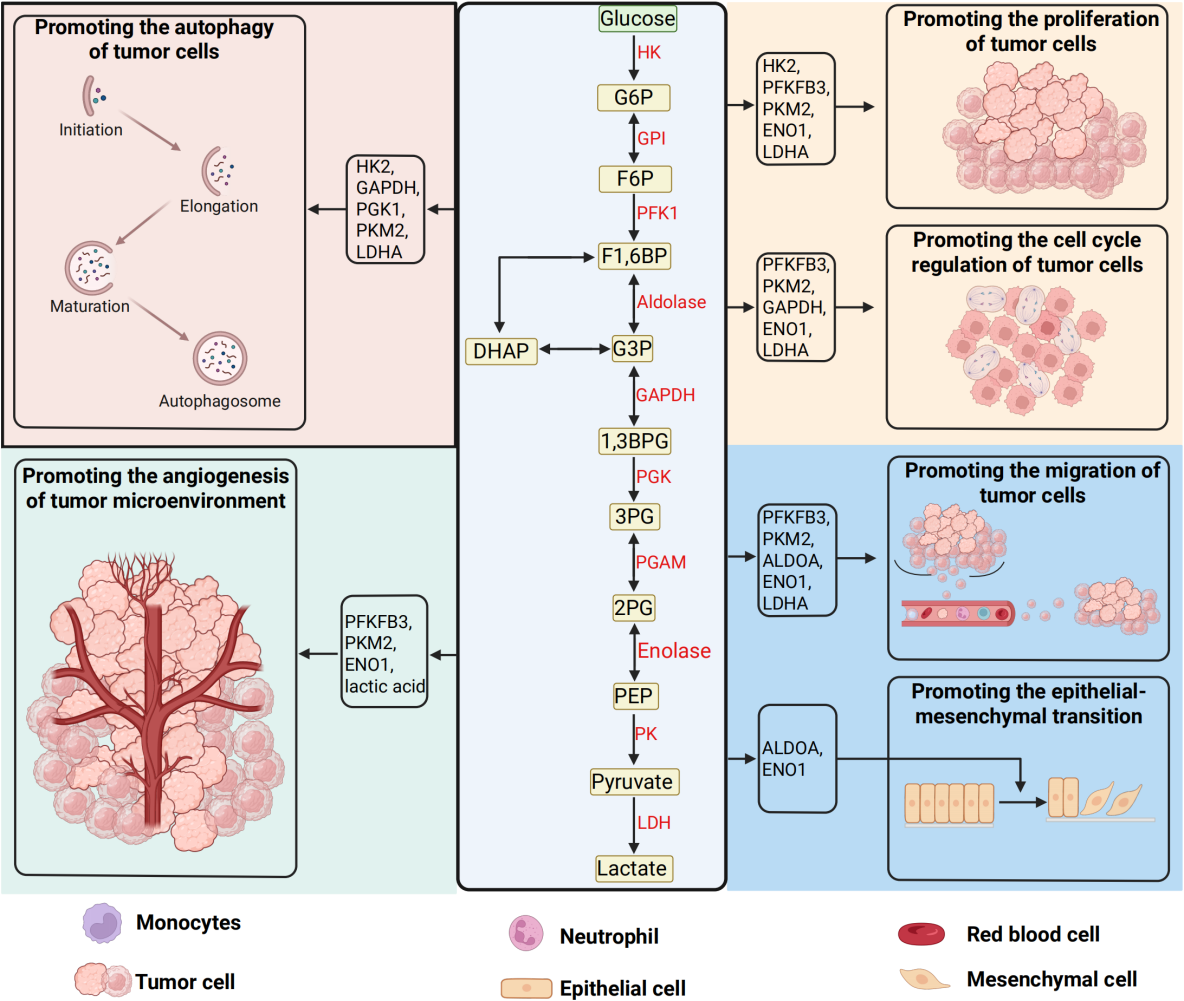
Glycolytic reprogramming and its oncogenic circuitry in tumor development. The glycolytic pathway, a hallmark of cancer metabolism, orchestrates the sequential breakdown of glucose into lactate under both hypoxic and normoxic conditions—a phenomenon known as the Warburg effect. This cytoplasmic cascade initiates with glucose uptake mediated by solute carrier transporters (SLC2A/GLUT family), which comprises 14 isoforms with distinct tissue-specific expression and regulatory roles in tumor biology. Intracellular glucose undergoes stepwise enzymatic processing: hexokinase (HK) traps glucose via phosphorylation, phosphofructokinase-1 (PFK-1) gates glycolytic flux through allosteric regulation, and pyruvate kinase (PK) catalyzes the terminal ATP-generating step to yield pyruvate. Under oxygen deprivation, lactate dehydrogenase (LDH) redirects pyruvate toward lactate production, sustaining NAD^+^ regeneration for continued glycolysis. Beyond energy production, glycolytic intermediates and enzymes directly engage in oncogenic signaling—HK-2 stabilizes mitochondrial membranes to inhibit apoptosis, PKM2 translocates to the nucleus as a transcriptional coactivator, and lactate acidifies the tumor microenvironment to drive immune evasion, extracellular matrix remodeling, and metastatic dissemination. These multifaceted interactions position glycolysis as a central hub for metabolic plasticity, epigenetic reprogramming, and therapeutic resistance in malignancies. (Created by biorender.com).

Beyond their canonical metabolic roles, glycolytic enzymes exert pleiotropic control over malignant phenotypes through both catalytic and non-catalytic mechanisms (77–79). HK2, frequently overexpressed in advanced tumors, binds mitochondrial voltage-dependent anion channels (VDACs) to evade apoptosis while enhancing glucose phosphorylation (80, 81). LDHA, a key hypoxia-responsive enzyme, not only maintains glycolytic flux but also generates lactate-a potent oncometabolite that acidifies the TME to stabilize HIF-1α, activate TGF-β signaling, and induce epithelial-mesenchymal transition (EMT) (82–85). Notably, lactate-mediated TME acidification directly impairs cytotoxic T lymphocyte (CTL) function while polarizing tumor-associated macrophages (TAMs) toward immunosuppressive M2 phenotypes (86, 87). Pyruvate kinase M2 (PKM2), the embryonic splice variant re-expressed in cancers, exhibits dynamic oligomeric regulation: tetrameric forms catalyze phosphoenolpyruvate-to-pyruvate conversion, whereas dimeric PKM2 translocates to the nucleus, serving as a transcriptional coactivator for HIF-1α, signal transducer and activator of transcription 3 (STAT3), and β-catenin to drive cell cycle progression and stemness (37, 88–94). Overall, glycolytic reprogramming and its oncogenic circuitry in tumor development was shown in **Figure 2**.

### 2.4 GLUTs: gatekeepers of tumor metabolism

The GLUT family represents the first bottleneck in tumor glycolytic dependency, with isoform-specific expression patterns dictating metabolic adaptability (46, 95). GLUT1 overexpression, driven by H. pylori-induced NF-κB activation in gastric carcinogenesis, correlates with advanced Tumor node metastasis classification(TNM) staging, venous invasion, and reduced 5-year survival in GC (96). Intriguingly, GLUT3 -typically restricted to neurons-becomes aberrantly expressed in therapy-resistant tumors, enabling glucose uptake under hypoglycemic TME conditions (97, 98). Clinical evidence reveals dynamic GLUT regulation during treatment: neoadjuvant chemotherapy downregulates GLUT4 in GC patients, coinciding with acquired chemoresistance through PI3K/AKT pathway activation (99, 100). Preclinical models demonstrate that dual targeting of GLUT1 and GLUT3 synergistically inhibits glycolytic flux and restores cisplatin sensitivity in refractory GC cells (101). These findings position GLUT isoform-specific inhibition as a promising strategy to circumvent metabolic adaptations underlying treatment failure.

## 3 Epigenetic orchestration of glycolytic reprogramming in gastric cancer

**Figure 3:**
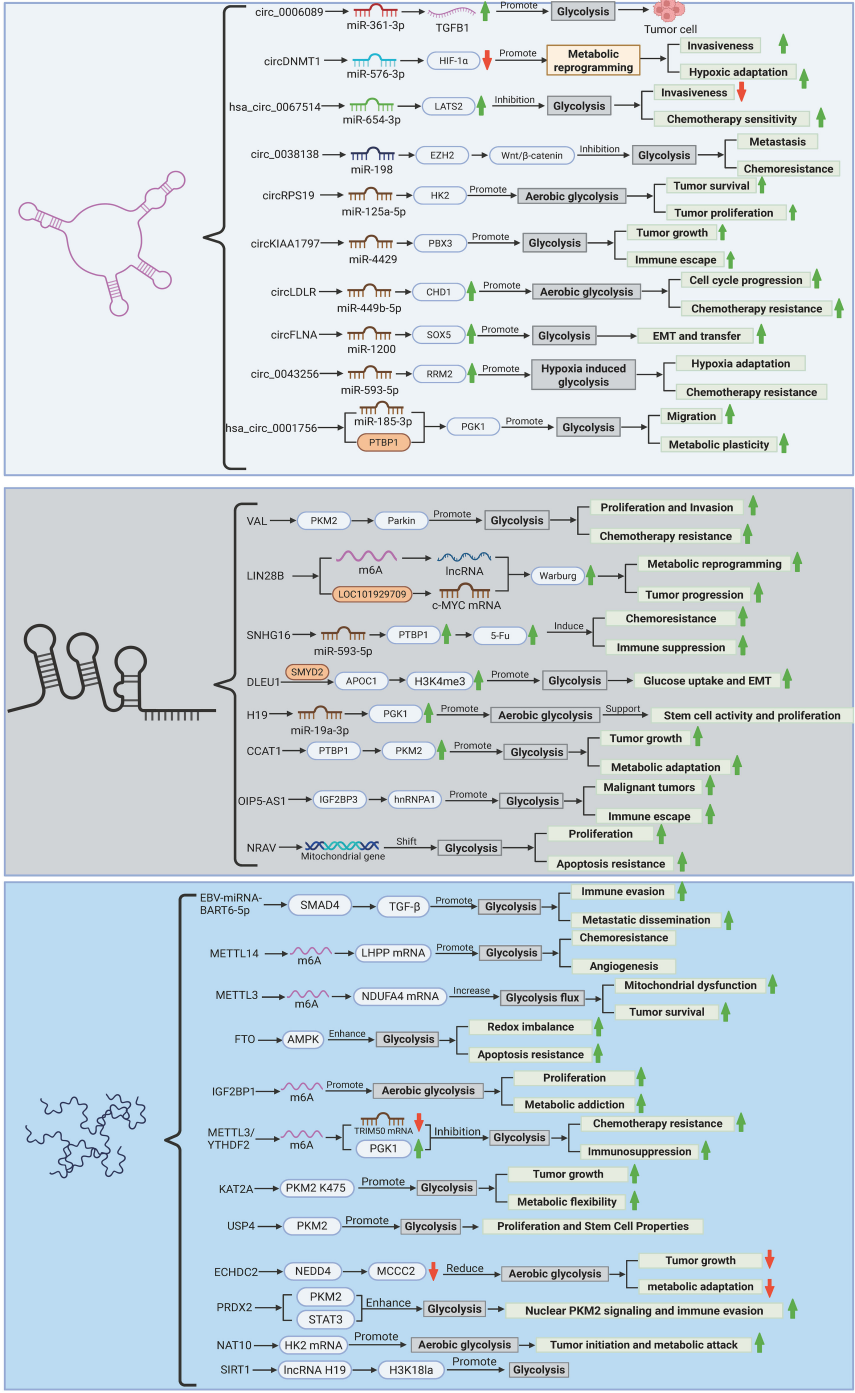
Epigenetic regulation of glycolytic reprogramming in gastric cancer. The figure illustrates the multilayered epigenetic control of glycolytic rewiring in gastric carcinogenesis. Non-coding RNAs (circRNAs, lncRNAs, and viral miRNAs) orchestrate metabolic adaptation through ceRNA networks, splicing regulation, and enzyme stabilization (e.g., circ_0006089/miR-361-3p/TGFB1; VAL/PKM2 ubiquitination). m6A epitranscriptomic modifications (METTL3/14, FTO, IGF2BP1) fine-tune glycolytic enzyme expression via mRNA stability and translation efficiency. Post-translational modifications (succinylation, deubiquitination, lactylation) dynamically regulate PKM2/HK2 activity and chromatin remodeling, linking glycolysis to oncogenic signaling. Hypoxia, nutrient stress, and viral integration converge to sustain a Warburg phenotype, driving proliferation, chemoresistance, and immune evasion, while highlighting potential therapeutic targets (e.g., METTL3 inhibitors, circRNA-directed siRNAs). (Created by biorender.com).

Gastric cancer (GC) progression is intricately tied to metabolic reprogramming, with dysregulated glycolytic flux—manifested as the Warburg effect—serving as a hallmark of tumor bioenergetics. Emerging evidence underscores the pivotal role of epigenetic mechanisms in orchestrating this metabolic shift through multilayered regulatory networks. Non-coding RNAs, including circRNAs and lncRNAs, dictate glycolytic adaptation via miRNA sponging, RNA splicing modulation, and epigenetic remodeling, while viral miRNAs further disrupt host metabolic checkpoints. Concurrently, dynamic m6A epitranscriptomic modifications fine-tune glycolytic enzyme expression through methyltransferase/eraser-mediated RNA methylation cycles. Post-translational modifications of metabolic kinases and transporters add another regulatory stratum, directly modulating enzyme stability and activity. These interconnected mechanisms collectively sustain metabolic plasticity, drive therapeutic resistance, and establish tumor-microenvironment crosstalk. The complexity of this epigenetic-metabolic interplay is systematically dissected in subsequent sections, with comprehensive regulatory hierarchies and molecular interactions detailed in **Figure 3** and **Table 1**. Elucidating these pathways unveils novel vulnerabilities for precision therapeutic targeting in GC.

**Table 1:** Epigenetic and transcriptional regulation of glycolysis in gastric cancer.

Regulation	Subcategory	Target	Key Regulatory Mechanism	Effect on Glycolysis	Biological Roles in GC	Reference
Non-coding RNA Networks	circRNA/miRNA axes	circ_0006089	Sponges miR-361-3p to upregulate TGFB1	Promotes glycolysis	Promotes tumor growth, angiogenesis, and metastasis	(102)
		circDNMT1	Sponges miR-576-3p to inhibit HIF-1α degradation	Promotes metabolic reprogramming	Enhances invasion and hypoxia adaptation	(103)
		hsa_circ_0067514	Sponges miR-654-3p to upregulate LATS2	Suppresses glycolysis	Inhibits invasion and improves chemosensitivity	(108)
		circ_0038138	Sponges miR-198 to stabilize EZH2, activating Wnt/β-catenin	Enhances glycolysis	Drives metastasis and chemoresistance	(104)
		circRPS19	Sponges miR-125a-5p to stabilize HK2 via USP7-mediated deubiquitination	Promotes aerobic glycolysis	Accelerates tumor proliferation and survival	(105)
		circKIAA1797	Sponges miR-4429 to upregulate PBX3	Enhances glycolysis	Promotes tumor growth and immune evasion	(109)
		circLDLR	Sponges miR-449b-5p to upregulate CHD1	Promotes aerobic glycolysis	Drives cell cycle progression and chemoresistance	(171)
		circFLNA	Sponges miR-1200 to upregulate SOX5	Enhances glycolysis	Facilitates EMT and metastasis	(172)
		circ_0043256	Sponges miR-593-5p to upregulate RRM2	Promotes hypoxia-induced glycolysis	Mediates hypoxia adaptation and chemotherapy resistance	(107)
		hsa_circ_0001756	Sponges miR-185-3p and binds PTBP1 to stabilize PGK1	Enhances glycolysis	Promotes migration and metabolic plasticity	(106)
	lncRNA Interactions	VAL	Binds PKM2 to block Parkin-mediated ubiquitination	Increases aerobic glycolysis	Enhances proliferation, invasion, and chemoresistance	(110)
		LIN28B	Stabilizes c-MYC mRNA via m6A recognition with lncRNA LOC101929709	Enhances Warburg effect	Drives metabolic reprogramming and tumor progression	(117)
		SNHG16	Sponges miR-506-3p to upregulate PTBP1	Mediates 5-Fu resistance	Induces chemoresistance and immune suppression	(116)
		DLEU1	Recruits SMYD2 to APOC1 promoter, increasing H3K4me3	Promotes glycolysis	Enhances glucose uptake and EMT	(113)
		H19	Sponges miR-19a-3p to upregulate PGK1	Enhances aerobic glycolysis	Supports stemness and proliferation	(115)
		CCAT1	Stabilizes PTBP1 to promote PKM2 splicing	Increases glycolysis	Drives tumor growth and metabolic adaptation	(111)
		OIP5-AS1	Binds IGF2BP3 (m6A-dependent) and stabilizes hnRNPA1 to activate PKM2 signaling	Promotes glycolysis	Enhances malignancy and immune escape	(112)
		NRAV	Suppresses mitochondrial genes to shift metabolism to glycolysis	Drives glycolytic shift	Accelerates proliferation and apoptosis resistance	(114)
	Viral miRNA Regulation	EBV-miRNA-BART6-5p	Targets SMAD4 to activate TGF-β signaling	Enhances glycolysis	Promotes immune evasion and metastatic dissemination	(118)
m6A Methylation		METTL14	Mediates m6A modification of LHPP mRNA to suppress its expression	Promotes glycolysis	Induces chemoresistance and angiogenesis	(119)
		METTL3	Stabilizes NDUFA4 mRNA via m6A to enhance glycolysis and oxidative metabolism	Increases glycolytic flux	Supports mitochondrial dysfunction and tumor survival	(120)
		FTO	Demethylates PRKAA1 (AMPK) mRNA to stabilize it	Enhances glycolysis	Drives redox imbalance and apoptosis resistance	(121)
		IGF2BP1	Recognizes m6A on c-MYC mRNA to stabilize it	Drives aerobic glycolysis	Promotes proliferation and metabolic addiction	(122)
		METTL3/YTHDF2	Degrade TRIM50 mRNA via m6A to upregulate PGK1	Inhibits glycolysis	TRIM50 loss enhances chemoresistance and immune suppression	(123)
Post-Translational Modifications	Enzyme Activity	KAT2A	Catalyzes PKM2 K475 succinylation to enhance its activity	Promotes glycolysis	Drives tumor growth and metabolic flexibility	(124)
	Ubiquitination/Deubiquitination	USP4	Deubiquitinates PKM2 to stabilize it	Enhances glycolysis	Supports proliferation and stemness	(125)
		ECHDC2	Recruits NEDD4 to degrade MCCC2, suppressing PKM2/GLUT1	Reduces aerobic glycolysis	Inhibits tumor growth and metabolic adaptation	(126)
		PRDX2	Binds and stabilizes PKM2, activating STAT3	Enhances glycolysis	Promotes nuclear PKM2 signaling and immune evasion	(136)
	Acetylation	NAT10	Acetylates HK2 mRNA (ac4C modification) to promote stability	Drives aerobic glycolysis	Supports tumor initiation and metabolic aggression	(127)
	Histone Lactylation	SIRT1	Downregulation increases H3K18la via lncRNA H19/glycolysis feedback	Enhances glycolysis	Links metabolic rewiring to epigenetic dysregulation	(128)

### 3.1 Non-coding RNA regulatory circuits

#### 3.1.1 circRNA-miRNA crosstalk in metabolic rewiring

Emerging evidence delineates circular RNA (circRNA)-mediated competitive endogenous RNA (ceRNA) networks as master regulators of glycolytic adaptation in GC. Oncogenic circRNAs exhibit tumor-promoting effects through miRNA sequestration and downstream target derepression. For example, hypoxia-elevated circ_0006089 (102) competitively binds miR-361-3p to activate Transforming Growth Factor Beta 1 (TGFB1) signaling, driving simultaneous enhancement of glycolytic flux, proliferative capacity, and angiogenic potential. Similarly, circDNMT1 (103) sustains HIF-1α protein stability by antagonizing miR-576-3p, thereby coordinating hypoxia-induced metabolic reprogramming with metastatic dissemination. Tumor-derived exosomes exploit this mechanism by packaging circ_0038138 (104), which sequesters miR-198 to relieve enhancer of zeste homolog 2 (EZH2) suppression, establishing a Wnt/β-catenin-dependent pro-metastatic niche through glycolysis-derived ATP provision.

Direct modulation of glycolytic enzymes occurs via circRNA scaffolds: circRPS19 (105) stabilizes HK2 through dual mechanisms involving miR-125a-5p sponging and USP7-mediated deubiquitination, while hsa_circ_0001756 (106) enhances phosphoglycerate kinase 1 (PGK1) expression via miR-185-3p neutralization and polypyrimidine tract-binding protein 1(PTBP1)-dependent mRNA stabilization. Metabolic vulnerabilities arise from circRNA-driven feedback loops, exemplified by hypoxia-inducible circ_0043256 (107) which couples ribonucleotide reductase M2 (RRM2) overexpression with enhanced glycolytic output, enabling chemoresistance through coordinated nucleotide/glycolytic metabolism.

Tumor-suppressive circRNAs demonstrate therapeutic potential through metabolic constraint. Restoration of hsa_circ_0067514 (108) reverses Warburg metabolism by liberating Large Tumor Suppressor Kinase 2(LATS2) from miR-654-3p-mediated repression, reinstating Hippo pathway-mediated growth control. Paradoxically, circKIAA1797 (109) exhibits compartment-specific regulation: intracellular accumulation promotes glycolysis via miR-4429/PBX3 axis activation, whereas exosomal depletion disrupts tumor-stroma metabolic coupling. These findings nominate circRNA-directed interventions as viable strategies to disrupt metabolic plasticity.

#### 3.1.2 LncRNA networks in metabolic adaptation

Long non-coding RNAs (lncRNAs) coordinate multilayered control over GC metabolism through epigenetic, post-transcriptional, and post-translational mechanisms. The lncRNA VAL (110) sustains PKM2 activity by obstructing Parkin-mediated ubiquitination, thereby maintaining aerobic glycolysis and stemness properties. Splicing regulation represents another axis of control: CCAT1 (111) interacts with PTBP1 to enforce PKM2 isoform switching, while OIP5-AS1 (112) stabilizes hnRNPA1 via m6A reader YTHDF2 recruitment, enhancing PKM2 mRNA nuclear export and translation.

Epigenetic reprogramming by lncRNAs directly impacts glycolytic gene expression. Deleted in Leukemia 1 (DLEU1) (113) recruits the histone methyltransferase SMYD2 to catalyze H3K4me3 deposition at the Apolipoprotein C1 (APOC1) promoter, activating this key regulator of GLUT trafficking. Genetic polymorphisms further modulate metabolic programming, as evidenced by the NRAV locus (114), where the rs6489786 Single Nucleotide Polymorphism (SNP) strengthens MEOX1/2 transcription factor binding to drive NRAV overexpression and mitochondrial complex I suppression.

Mechanistic diversity extends to miRNA sponging: H19 (115) and Small Nucleolar RNA Host Gene 16(SNHG16) (116) sequester miR-675-5p and miR-519a-3p respectively, derepressing PGK1 and HK1/2 to confer 5-FU resistance. Synergistic regulation occurs through m6A-mediated mRNA stabilization, exemplified by LIN28B (117)/LOC101929709 complexes that enhance cellular myelocytomatosis oncogene (c-MYC) transcript stability via YTHDF1 recruitment, amplifying glycolytic enzyme transcription. Emerging therapeutic modalities include lipid nanoparticle-encapsulated siRNAs targeting VAL/CCAT1, dCas9-KRAB-mediated epigenetic silencing of DLEU1/NRAV loci, and miRNA mimics to counteract oncogenic sponge activity.

#### 3.1.3 Viral miRNA subversion of host metabolism

Epstein-Barr virus (EBV) orchestrates miRNA-mediated metabolic hijacking in EBV-associated gastric cancer (EBVaGC). The viral miRNA BART6-5p (118) destabilizes SMAD4 mRNA through direct 3’UTR targeting, activating TGF-β/SMAD signaling to drive epithelial-mesenchymal transition (EMT) while concurrently upregulating hexokinase 2 (HK2) and lactate dehydrogenase A (LDHA) via indirect mechanisms. This dual metabolic-transcriptional reprogramming fosters an aggressive tumor phenotype, rendering it susceptible to combinatorial strategies involving BART6-5p antagomirs and LDHA inhibitors. Outstanding questions persist regarding how EBV miRNAs coordinate with host immune metabolic networks—particularly the regulation of PD-L1/IDO1 within immune-evasive niches.

### 3.2 m6A epitranscriptomic control of glycolytic circuits

Dynamic RNA m6A methylation fine-tunes glycolytic enzyme expression through writer/eraser/reader coordination. Methyl transferase-like 14 (METTL14)-mediated m6A deposition on LHPP transcripts suppresses their translation (119), releasing GSK3β-mediated inhibition of HIF1α to drive GLUT1/LDHA transcription. Conversely, METTL3 (120) stabilizes NDUFA4 mRNA via YTHDF1 recognition, enabling paradoxical co-activation of glycolysis and residual OXPHOS in treatment-resistant clones. Demethylase FTO (121) sustains energy homeostasis by erasing m6A marks from PRKAA1 mRNA, preserving its stability under glucose deprivation.

Downstream effectors integrate metabolic and oncogenic signaling: IGF2BP1 (122) recognizes m6A-modified c-MYC transcripts to enhance LDHA/HK2 expression, establishing a self-reinforcing loop through c-MYC-driven mTOR activation. Novel regulatory layers include METTL3/YTHDF2-mediated degradation of TRIM50 mRNA (123), which elevates PGK1 protein levels by reducing E3 ligase-mediated ubiquitination. Clinically, small-molecule inhibitors targeting METTL3 and IGF2BP1 show synergistic effects with PD-1 blockade in preclinical models, suggesting combined metabolic-immunotherapeutic potential.

### 3.3 Post-translational regulation of metabolic enzymes

Dynamic post-translational modifications (PTMs) serve as metabolic rheostats in GC. Lysine succinylation by KAT2A (124) enhances PKM2 tetramerization and activity, while USP4-mediated deubiquitination prolongs PKM2 half-life (125). Counter-regulatory mechanisms involve ECHDC2-dependent recruitment of NEDD4 to degrade methylcrotonoyl-CoA carboxylase 2 (MCCC2) (126), indirectly suppressing PKM2/GLUT1 through acetyl-CoA depletion.

Nutrient-responsive PTMs integrate environmental cues with enzyme activity. Under glucose-replete conditions, NAT10 catalyzes ac4C acetylation of HK2 mRNA (127), boosting translation efficiency. Conversely, glucose deprivation triggers NAT10 degradation and HK2 downregulation, exemplifying nutrient-epitranscriptome coupling. Novel histone lactylation links glycolytic output to chromatin state (128), where lactate-derived H3K18la modifications activate oncogenic lncRNA H19 transcription, while SIRT1 downregulation perpetuates this feedforward cycle through impaired deacetylation.

## 4 Metabolic enzyme networks as actionable targets in GC

Metabolic enzyme networks constitute actionable therapeutic targets in GC, driving tumor progression through isoform-specific regulation, dynamic structural modulation, and auxiliary pathway exploitation. Hexokinase isoforms (HK1, HK2, HKDC1) exhibit divergent oncogenic mechanisms, from mitochondrial-nuclear crosstalk to chemoresistance-linked redox cycling. PKM2 functions as a pleiotropic metabolic rheostat, integrating glycolytic flux with proliferative and inflammatory signaling via phosphorylation-dependent oligomerization and ubiquitination dynamics. Beyond canonical enzymes, auxiliary players such as PGAM1 and ALDOB expand targetable space by coupling metabolic rewiring with immune evasion and DNA damage response. These networks are exploitable through isoform-specific inhibitors, allosteric modulators, and RNA-based therapeutics, supported by advanced metabolic imaging for real-time therapeutic monitoring. The molecular hierarchies and therapeutic strategies governing these enzyme networks are systematically cataloged in **Table 2**, providing a roadmap for precision targeting of GC metabolic vulnerabilities.

**Table 2:** Key glycolytic enzymes as therapeutic targets in gastric cancer.

Enzyme	Regulatory Mechanism	Key Findings	Biological Roles	Therapeutic Potential	Reference
Hexokinase Family					
HK2	uMtCK activates JNK-MAPK/JUN pathway to upregulate HK2	uMtCK overexpression correlates with advanced GC stages and poor prognosis.	Promotes glycolysis, migration, invasion, and liver metastasis.	Targeting uMtCK/HK2 axis may suppress metastasis.	(129)
HK2	PDLIM1 binds HK2 to enhance Warburg effect via Wnt/β-catenin	PDLIM1 upregulation increases glycolysis and chemoresistance.	Drives proliferation, EMT, and metabolic adaptation.	PDLIM1/HK2 inhibition reverses chemoresistance.	(130)
HK1	DDX24 binds HK1 mRNA to enhance its transcription	DDX24 overexpression promotes glucose uptake and lactate production.	Enhances proliferation, migration, and invasion.	Targeting DDX24/HK1 suppresses glycolytic reprogramming.	(131)
HKDC1	HKDC1 mediates TGF-β/Smad2/EMT axis in H. pylori-induced GC	HKDC1 is upregulated in H. pylori-related GC and promotes EMT.	Facilitates metastasis and chemoresistance.	HKDC1 knockdown sensitizes GC cells to cisplatin/oxaliplatin.	(133)
HKDC1	HKDC1 overexpression enhances glycolysis and chemoresistance	HKDC1 correlates with lymph node metastasis and advanced TNM stages.	Promotes tumorigenesis and EMT.	HKDC1 silencing reduces tumor growth and improves chemotherapy response.	(132)
Pyruvate Kinase M2 (PKM2)					
PKM2	ARRB1 binds PKM2 to inhibit tetramerization, shifting metabolism to glycolysis	ARRB1 overexpression drives Warburg effect and proliferation.	Links metabolic reprogramming to cell cycle progression.	PKM2 activator DASA-58 reverses ARRB1-driven glycolysis.	(134)
PKM2	VAL blocks Parkin-mediated PKM2 ubiquitination to stabilize PKM2	VAL upregulation increases PKM2 activity and malignancy.	Enhances aerobic glycolysis, invasion, and chemoresistance.	Targeting VAL/PKM2 disrupts glycolytic addiction.	(110)
PKM2	CCAT1 stabilizes PTBP1 to promote PKM2 splicing (PKM1→PKM2)	CCAT1 overexpression enhances PKM2-dependent glycolysis.	Drives tumor growth and immune evasion.	CCAT1/PTBP1/PKM2 axis inhibition suppresses GC progression.	(111)
PKM2	LHX9 transcriptionally activates PKM2 in GC stem cells	LHX9/PKM2 axis induces glycolytic reprogramming in GC stem cells.	Maintains stemness and promotes metastasis.	Targeting LHX9/PKM2 may eliminate cancer stem cells.	(137)
PKM2	SHP2 dephosphorylates and activates PKM2, forming SHP2/PKM2/AMPK feedback loop	SHP2 overexpression correlates with poor prognosis and chemoresistance.	Enhances glycolysis and proliferation.	SHP2 inhibitor SHP099 synergizes with cisplatin.	(135)
PKM2	PRDX2 stabilizes PKM2 by inhibiting ubiquitination, activating STAT3	PRDX2 upregulation enhances nuclear PKM2-STAT3 signaling.	Promotes immune evasion and metabolic adaptation.	PRDX2/PKM2/STAT3 axis is a therapeutic vulnerability.	(136)
Other Enzymes					
PGAM1	PGAM1 overexpression activates glycolysis and immune pathways	PGAM1 correlates with aggressive tumor features (high grade, TNM stage).	Links glycolysis to immunosuppression.	PGAM1 inhibitors may reverse immune suppression.	(138)
ALDOB	ALDOB downregulation in GC promotes chemoresistance	ALDOB overexpression suppresses proliferation and enhances drug sensitivity.	Acts as a tumor suppressor by inhibiting glycolysis.	ALDOB restoration sensitizes GC to chemotherapy.	(139)
PDK4	TOP1MT loss upregulates PDK4 to enhance glycolysis	TOP1MT deletion promotes glycolysis and metastasis.	Drives metabolic plasticity and EMT.	PDK4 inhibitors (e.g., dichloroacetate) suppress metastasis.	(140)
PGK1	hsa_circ_0001756 stabilizes PGK1 via PTBP1 and miR-185-3p sponging	hsa_circ_0001756 upregulation correlates with tumor stage and size.	Enhances glycolysis, migration, and metabolic plasticity.	Targeting hsa_circ_0001756/PGK1 inhibits GC progression.	(106)

### 4.1 HK isoform-specific regulation

The HK family governs glycolytic entry points with isoform-specific roles in gastric carcinogenesis. HK2, the predominant isoform, is regulated through mitochondrial-nuclear crosstalk: ubiquitous mitochondrial creatine kinase (uMtCK) (129) activates JNK-MAPK/JUN signaling to drive HK2 transcription, establishing a liver-metastatic metabolic signature. Concurrently, cytoskeletal protein PDZ and LIM Domain Protein 1(PDLIM1) (130) physically interacts with HK2 to enhance catalytic efficiency while activating Wnt/β-catenin signaling - a dual mechanism creating metabolic-proliferative synergy. Nutrient-sensitive regulation is evidenced by PDLIM1 downregulation under glucose deprivation, suggesting adaptive rewiring capacity.

HK1 exhibits context-dependent oncogenicity through RNA helicase DEAD-box Helicase 24(DDX24) -mediated mRNA stabilization (131), promoting lactate-driven invasion in diffuse-type GC. The recently characterized Hexokinase Domain Containing 1(HKDC1) isoform demonstrates unique pathogenetic roles: its overexpression correlates with H. pylori-induced EMT via TGF-β/Smad2 activation (132, 133), while chemoresistance-linked HKDC1 upregulation (132) enables cisplatin evasion through glutathione redox cycling. Spatial transcriptomics reveals HKDC1 enrichment at invasive fronts, suggesting roles in metabolic adaptation during dissemination.

Therapeutic opportunities emerging from HK isoform biology include the development of uMtCK inhibitors to disrupt HK2/JNK-mediated metastatic programming, PDLIM1-targeted proteolysis-targeting chimeras (PROTACs) for simultaneous HK2 catalytic inhibition and Wnt pathway blockade, and HKDC1-specific locked nucleic acid (LNA) gapmers designed to reverse EMT in advanced GC. These targeted approaches are complemented by precision diagnostic strategies, where integration of Fluorine-18 Fluorodeoxyglucose Positron Emission Tomography (18F-FDG-PET) metabolic imaging with HK isoform immunohistochemical profiling enables patient stratification for HKDC1-directed therapies in Helicobacter pylori-positive cohorts, particularly those exhibiting chemoresistance and metastatic progression.

### 4.2 PKM2 as a metabolic rheostat

PKM2 serves as a nodal integrator of metabolic plasticity through dynamic structural regulation. β-arrestin 1 (ARRB1) (134) enforces glycolytic commitment by locking PKM2 in inactive dimers, while Src Homology 2 Domain-Containing Protein Tyrosine Phosphatase 2 (SHP2) phosphatase (135) activates PKM2 through Y105 dephosphorylation, creating an AMPK-mediated feedforward loop. Compartment-specific functions emerge: cytoplasmic ARRB1 suppresses PKM2 activity, whereas nuclear ARRB1 co-activates E2F1 to drive proliferation - a dichotomy underscoring PKM2’s pleiotropic roles.

PKM2 stability is regulated by ubiquitination. For example, lncRNA VAL prevents Parkin-mediated degradation, and PRDX2 stabilizes PKM2 while enhancing STAT3 co-activation (110, 136) This PRDX2-PKM2-STAT3 axis creates an inflammatory-metabolic circuit, with STAT3 reciprocally inducing PRDX2 transcription. Transcriptional control is mediated by LHX9-driven PKM2 promoter activation in stem-like cells (137), and CCAT1-enhanced PTBP1 splicing factor activity favoring PKM2 isoform retention (111).

Emerging therapeutic strategies targeting PKM2 regulation layers encompass allosteric activators such as DASA-58, which counteract ARRB1-induced dimerization to restore metabolic balance, and SHP2 inhibitors including SHP099 that disrupt kinase-metabolic crosstalk by blocking PKM2 dephosphorylation. Concurrently, thioredoxin-mimetic compounds designed to neutralize PRDX2 activity show promise in restoring Parkin-mediated PKM2 ubiquitination, thereby destabilizing the PRDX2-PKM2-STAT3 inflammatory-metabolic axis. Rational combination therapies, exemplified by DASA-58 paired withSTAT3 inhibitors, demonstrate enhanced efficacy in preclinical models by overcoming compensatory signaling pathways. To optimize therapeutic precision, metabolic flux analysis employing hyperpolarized ¹³C-pyruvate tracers provides real-time quantification of PKM2 modulator effects on glycolytic activity, enabling dynamic dose adjustment based on tumor-specific metabolic vulnerabilities.

### 4.3 Auxiliary enzymes expanding targetable space

Beyond canonical targets, auxiliary enzymes offer novel intervention points. Phosphoglycerate mutase 1 (PGAM1) (138) drives metabolic-immune crosstalk by activating IL-6/STAT3 signaling - a dual mechanism promoting both glycolytic flux and PD-L1-mediated immunosuppression. Contrastingly, aldolase B (ALDOB) (139) exhibits tumor-suppressive metabolic functions: its downregulation in intestinal-type GC impairs 5-FU-induced DNA damage response, while re-expression restores chemosensitivity through p21-mediated cell cycle arrest. Mitochondrial-nuclear coordination is disrupted by Topoisomerase 1 Mitochondrial (TOP1MT) loss (140), which upregulates Pyruvate Dehydrogenase Kinase 4(PDK4) to shunt pyruvate into lactate production. PDK4 inhibitors reverse this Warburg shift while suppressing lung metastasis in PDX models. CircRNA-mediated regulation emerges through hsa_circ_0001756 (106), which stabilizes PGK1 via miR-185-3p sponging and PTBP1-dependent mRNA stabilization - a mechanism maintaining metabolic heterogeneity in hypoxic niches.

Translational development priorities encompass PGAM1 allosteric inhibitors such as PGAMi to disrupt the IL-6/STAT3 immunosuppressive axis, ALDOB mRNA stabilizers derived from branched-chain amino acid analogs to restore chemosensitivity in intestinal-type GC, and PDK4-targeted metabolic radiosensitizers designed to enhance radiation efficacy by reversing Warburg-mediated redox adaptation. These therapeutic innovations are synergistically supported by advanced functional imaging techniques, particularly 18F-fluorothymidine (18F-FLT) PET, which enables non-invasive real-time monitoring of PGAM1 inhibitor target engagement and subsequent DNA synthesis modulation, facilitating precision dose optimization in heterogeneous tumor ecosystems.

## 5 Metabolic signaling networks in GC pathobiology

**Figure 4:**
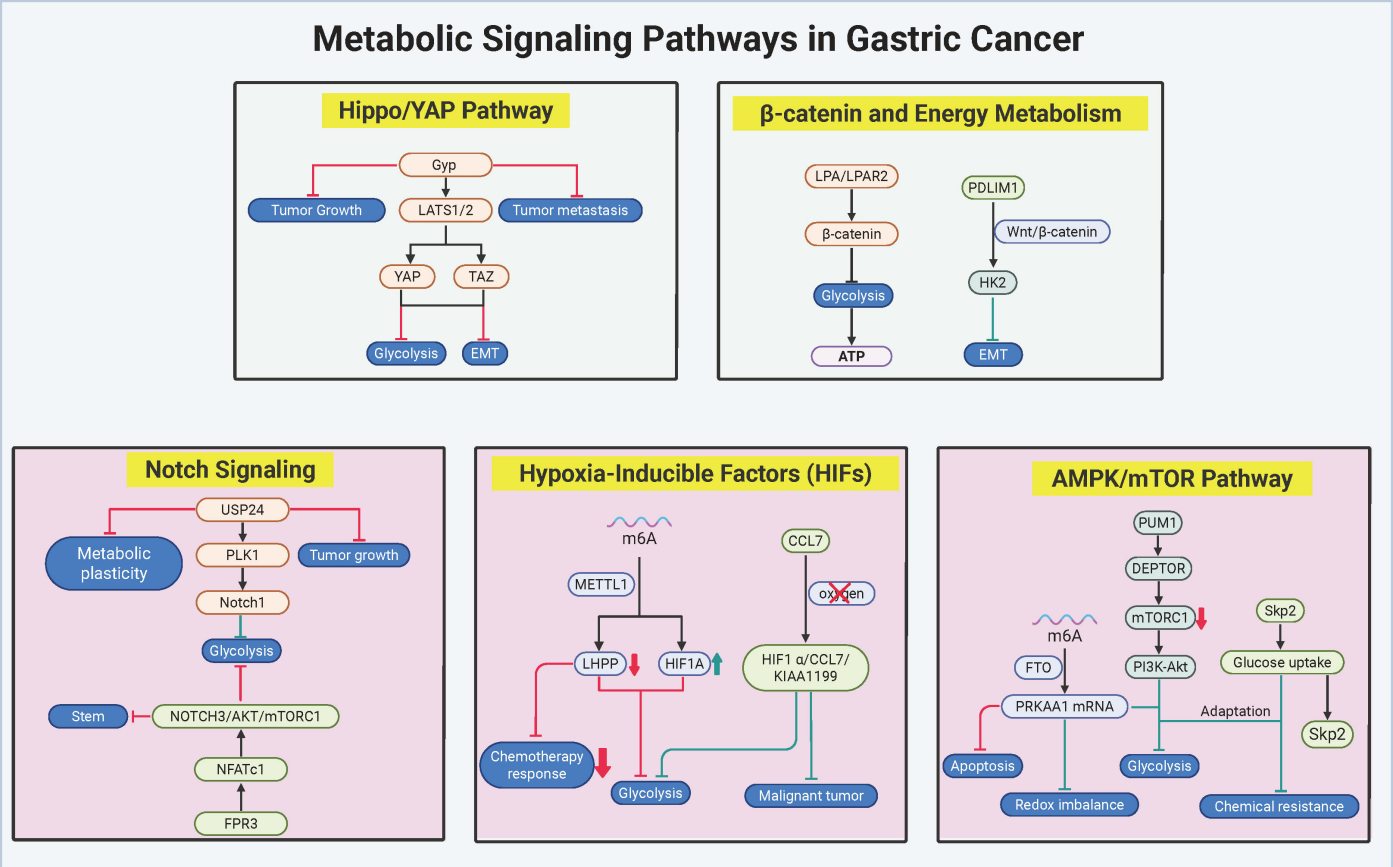
Metabolic signaling networks in gastric cancer pathobiology. The figure delineates the pivotal metabolic-oncogenic crosstalk underpinning therapeutic resistance and malignant progression in gastric cancer (GC), emphasizing interconnected core pathways. The AMPK/mTOR and Hippo/YAP axes synergistically coordinate metabolic adaptation through Skp2-mediated glucose-lactate cycling and YAP/TAZ-driven glycolytic transcription, while hypoxic signaling perpetuates stemness and immune evasion via HIF1α-CCL7-KIAA1199 feedback loops and METTL14-dependent m6A methylation. Concurrently, β-catenin and Notch pathways bridge mitochondrial-glycolytic coupling through LPAR2/PDLIM1 signaling, with USP24/PLK1/Notch1 enforcing subtype-specific metabolic rewiring in intestinal-type GC. Therapeutic vulnerabilities are exemplified by thioridazine-induced Skp2 degradation to disrupt glucose-lactate cycling, Ginkgolide-mediated YAP/TAZ inhibition achieving 76% tumor suppression in PDX models, and combinatorial targeting of LPAR2/PDLIM1 or USP24/PI3K to counteract resistance mechanisms. Molecular interactions, validated through preclinical studies and spatial transcriptomic profiling, underscore the clinical potential of stratifying patients based on metabolic signaling network activity. (Created by biorender.com).

Metabolic signaling networks in gastric cancer (GC) function as dynamic regulatory hubs, integrating oncogenic drivers with bioenergetic reprogramming to fuel malignant progression and therapy resistance. Core pathways—including the AMPK/mTOR axis, hypoxic signaling, β-catenin cascades, Notch circuits, and Hippo/YAP effectors—exhibit bidirectional crosstalk with metabolic enzymes, enabling adaptive survival under fluctuating nutrient availability and therapeutic pressure. These networks operate through multilayered mechanisms: epitranscriptomic RNA methylation, nutrient-sensitive protein stabilization, and subtype-specific epigenetic rewiring, which collectively synchronize glycolytic flux with programs governing proliferation, stemness, and immune evasion. Therapeutic vulnerabilities emerge from pathway interdependencies, as exemplified by combinatorial targeting of metabolic-transcriptional feedback loops and spatial regulation of enzyme-transporter complexes. The molecular architecture and hierarchical interactions of these signaling-metabolic axes are systematically mapped in **Figure 4**, with key regulatory nodes and therapeutic strategies cataloged in **Table 3**. Deciphering this circuitry provides a framework for precision interventions that disrupt metabolic adaptation while counteracting compensatory oncogenic signaling.

**Table 3:** Metabolic signaling pathways in gastric cancer.

Signaling Pathway	Key Regulator	Mechanism	Functional Impact	Reference
AMPK/mTOR Pathway				
PRKAA1 (AMPK)	FTO-mediated m6A modification stabilizes PRKAA1 mRNA	Enhances glycolysis and redox imbalance; inhibits apoptosis.	Correlates with poor prognosis; potential target for metabolic reprogramming.	(121)
PUM1	Stabilizes DEPTOR to inhibit mTORC1, activating PI3K-Akt	Promotes glycolysis and tumor progression.	High PUM1 expression linked to recurrence and metastasis.	(52)
Skp2	Skp2/AKT/mTOR/GLUT1 axis drives glucose uptake	Mediates chemoresistance and glycolytic adaptation.	Thioridazine targeting Skp2 synergizes with trastuzumab/lapatinib.	(141)
Hypoxia-Inducible Factors (HIFs)				
LHPP	METTL14-mediated m6A modification suppresses LHPP, activating HIF1A	Inhibits glycolysis and metastasis.	Low LHPP expression predicts poor chemotherapy response.	(119)
CCL7	HIF1α/CCL7/KIAA1199 axis under hypoxia	Enhances glycolysis and hypoxia-induced malignancy.	CCL7 inhibition reverses hypoxia-driven tumor aggression.	(142)
β-catenin and Energy Metabolism				
LPA/LPAR2	Activates β-catenin nuclear translocation	Increases ATP production via glycolysis/OXPHOS.	LPAR2 blockade suppresses metastasis and energy metabolism.	(143)
PDLIM1	Binds HK2 to enhance Warburg effect via Wnt/β-catenin	Drives proliferation and EMT.	PDLIM1 overexpression correlates with advanced TNM stages.	(130)
Notch Signaling				
USP24	Stabilizes PLK1 to activate Notch1	Enhances glycolysis and proliferation.	USP24 silencing inhibits tumor growth and metabolic plasticity.	(144)
FPR3	Inhibits NFATc1 nuclear translocation, downregulating NOTCH3/AKT/mTORC1	Reduces glycolysis and stemness.	FPR3 overexpression suppresses tumor progression; potential therapeutic agonist.	(145)
Hippo/YAP Pathway				
Gyp (Ginkgetin)	Activates LATS1/2 to phosphorylate YAP and degrade TAZ	Suppresses glycolysis and EMT.	Gyp inhibits tumor growth and metastasis; synergizes with Hippo-targeted therapies.	(146)

The intricate crosstalk between metabolic reprogramming and oncogenic signaling in GC drives therapeutic resistance and malignant progression through multilayered regulatory circuits. Central to this interplay is the AMPK/mTOR axis, where the FTO-PRKAA1 axis (121) stabilizes AMPK’s catalytic subunit PRKAA1 via m6A demethylation, paradoxically activating glycolytic flux while maintaining redox homeostasis under nutrient deprivation. This pro-survival adaptation diverges from AMPK’s classical energy-sensing role, implicating non-canonical HIF1α-mediated signaling. Simultaneously, the RNA-binding protein PUM1 (52) stabilizes DEPTOR mRNA to suppress mTORC1 activity, yet activates compensatory PI3K-Akt signaling—a dual mechanism enabling tumor proliferation during metabolic stress. The Skp2-AKT/mTOR axis (141) further integrates growth factor signaling with glucose metabolism, where Skp2 enhances GLUT1-mediated uptake and LDHA activation, creating a self-reinforcing glucose-lactate cycle. Preclinical validation using thioridazine, an antipsychotic that degrades Skp2, demonstrates synergistic suppression of HER2^+^ GC growth in patient-derived xenograft (PDX) models when combined with trastuzumab, underscoring the therapeutic value of targeting metabolic-growth pathway intersections.

Hypoxic signaling pathways shape GC metabolism through bidirectional epigenetic-metabolic coupling. METTL14-mediated m6A methylation (119) silences the tumor suppressor LHPP, releasing GSK3β to degrade HIF1α and attenuate glycolysis. Conversely, hypoxia-induced HIF1α (142) transcriptionally activates chemokine C-C Motif Chemokine Ligand 7(CCL7), establishing a HIF1α-CCL7-KIAA1199 feedforward loop that amplifies glycolytic output and EMT. Genetic ablation of CCL7 disrupts hypoxia-driven cancer stemness, revealing its critical role in metabolic plasticity. Therapeutic strategies combining LHPP agonists, CCL7-neutralizing antibodies, and m6A writer inhibitors show promise in preclinical models, with single-cell spatial transcriptomics emerging as a tool to map hypoxic niche-specific vulnerabilities.

The β-catenin pathway serves as a metabolic-proliferative nexus in GC. Lysophosphatidic acid (LPA) signaling through LPAR2 (143) activates β-catenin nuclear translocation, upregulating c-MYC and Cyclin D1 while coordinating mitochondrial OXPHOS and glycolysis for rapid ATP generation. Pharmacological LPAR2 antagonists concurrently inhibit β-catenin signaling and metabolic activation, demonstrating dual therapeutic efficacy. Spatial regulation is exemplified by PDLIM1 (130), which anchors HK2 at mitochondria to stabilize β-catenin by physically blocking GSK3β-mediated degradation—a mechanism disrupted under glucose deprivation, revealing nutrient-sensitive decoupling of metabolic and proliferative signaling. Combinatorial targeting of LPAR2 and PDLIM1 using small-molecule inhibitors, alongside CRISPR/dCas9-mediated epigenetic editing of β-catenin target genes, may synergistically suppress β-catenin-driven malignancy.

Notch signaling exhibits subtype-specific metabolic regulation in GC. The USP24/PLK1/Notch1 axis (144) stabilizes PLK1 via USP24-mediated deubiquitination, phosphorylating Notch1 to activate HES1/HEY1-driven glycolysis and cell cycle progression. Clinically, USP24 overexpression correlates with 5-FU resistance and reduced survival. In contrast, the FPR3/NFATc1/NOTCH3 axis (145) exerts tumor-suppressive effects in diffuse-type GC by modulating calcium flux: FPR3 activation restricts NFATc1 nuclear translocation, downregulating NOTCH3-AKT/mTOR signaling and suppressing stemness. Therapeutic exploitation includes USP24 inhibitors combined with PI3K inhibitors in intestinal-type GC, versus FPR3 agonists in diffuse-type tumors, guided by single-cell EMT and immune subtype profiling.

The Hippo/YAP pathway emerges as a master metabolic regulator. Ginkgolide (146), a natural compound, activates LATS1/2 to phosphorylate YAP and degrade TAZ, suppressing YAP/TAZ-mediated transcription of GLUT1 and LDHA. In PDX models, Gyp reduces tumor volume by 76% and FDG-PET SUVmax by 82%, with nuclear YAP phosphorylation serving as a predictive biomarker for therapeutic response. Clinically relevant combinatorial regimens pairing YAP/TAZ inhibitors with mTOR blockers enhance efficacy while mitigating compensatory signaling.

## 6 Metabolic vulnerabilities and therapeutic resistance in GC

**Figure 5:**
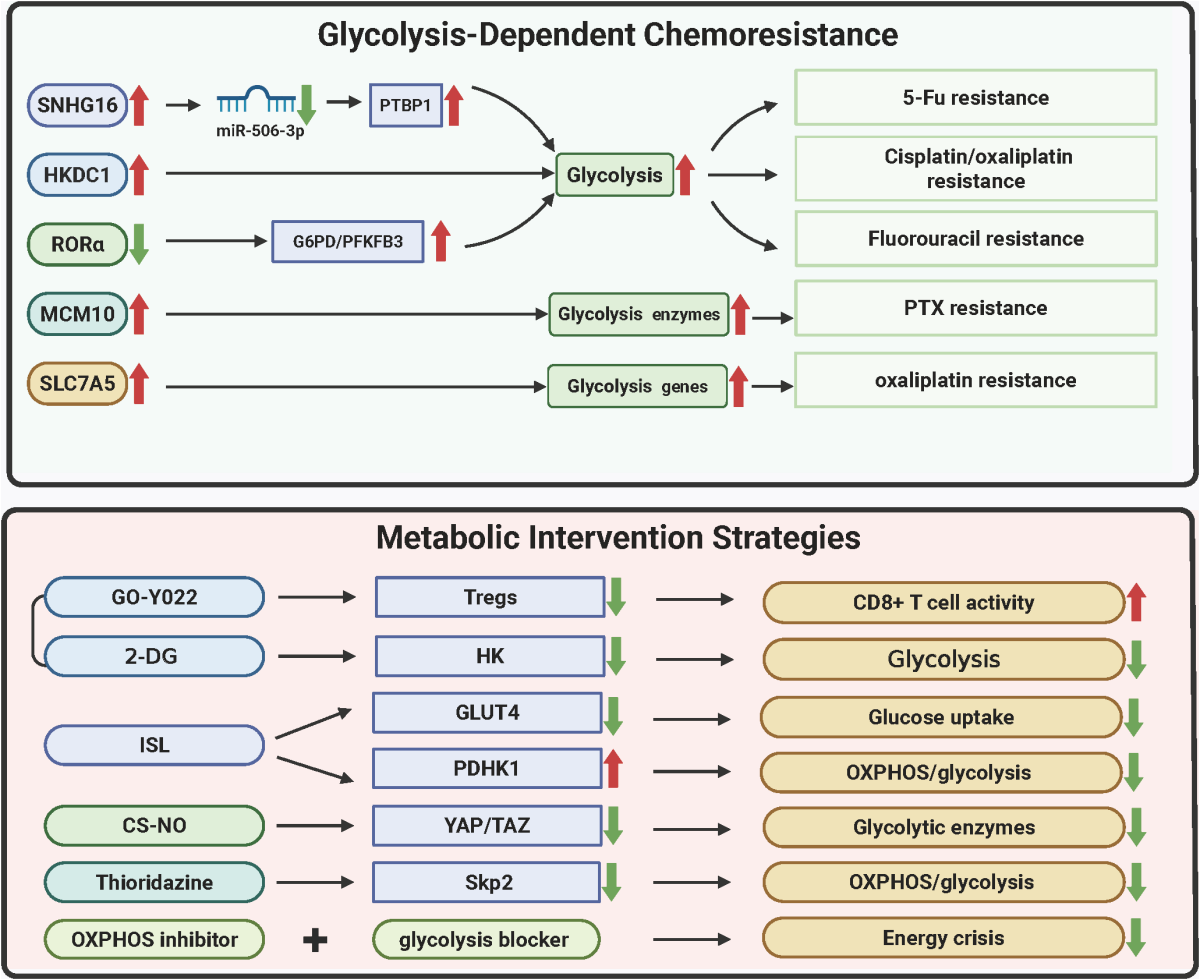
Metabolic vulnerabilities and therapeutic resistance in gastric cancer. The figure delineates metabolic adaptations driving chemoresistance in GC and corresponding therapeutic strategies. Glycolytic reprogramming via SNHG16/PTBP1 stabilizes glycolytic enzyme transcripts, conferring 5-FU resistance (58% tumor reduction with SNHG16 LNA inhibitors). HKDC1 and SLC7A5 couple glycolysis/OXPHOS and redox adaptation to promote cisplatin/oxaliplatin resistance, reversed by SLC7A5 silencing (62% lactate reduction). MCM10-mediated mitochondrial HK2 translocation sustains stemness under paclitaxel pressure, mitigated by nanoparticle-delivered siRNA (CSC reduction from 23% to 6%). RORα loss enhances fluorouracil resistance via PPP activation, while GO-Y022 + 2-DG disrupts immunosuppressive niches (82% regression via Treg suppression and CD8+ T-cell infiltration). Nanodelivery systems (e.g., CS-NO) degrade HK2/LDHA (89% lactate decrease) and inhibit metastasis, whereas thioridazine repurposing synergizes with trastuzumab (59% complete response). Dual glycolysis/OXPHOS inhibition induces necroptosis (73% disease control) monitored via hyperpolarized 13C-MRSI, highlighting metabolic imaging-guided precision therapy. (Created by biorender.com).

Metabolic vulnerabilities in GC converge with chemoresistance through dynamic adaptations in glycolysis, redox balancing, and mitochondrial-nuclear crosstalk. Glycolytic drivers such as lncRNA SNHG16 and HKDC1 rewire energy metabolism to sustain drug efflux and DNA repair, while stemness reprogramming via MCM10 and RORα loss couples metabolic plasticity with apoptotic evasion. Therapeutic strategies targeting these axes exploit multimodal approaches—nanoparticle-mediated enzyme degradation, metabolic-immune niche disruption, and pan-pathway inhibition—to induce synthetic lethality. Synergistic regimens combining OXPHOS/glycolysis blockade or HDAC inhibitors with metabolic modulators demonstrate rapid efficacy, validated by advanced imaging biomarkers. The molecular hierarchies of these resistance mechanisms and corresponding therapeutic interventions are systematically mapped in **Figure 5** and **Table 4**, providing a blueprint for overcoming GC’s adaptive metabolic resilience through precision targeting.

**Table 4:** Metabolic vulnerabilities and chemoresistance in gastric cancer.

Metabolic vulnerabilities	Target/Strategy	Mechanism	Key Findings	Reference
Glycolysis-Dependent Chemoresistance				
SNHG16/PTBP1 axis	lncRNA SNHG16/miR-506-3p/PTBP1 axis	SNHG16 upregulation suppresses miR-506-3p, leading to PTBP1 overexpression and enhanced glycolysis, driving 5-FU resistance.	Blocking the SNHG16/PTBP1 axis reduces tumor growth in 5-FU-resistant xenografts.	(116)
HKDC1	HKDC1-mediated glycolysis and EMT	HKDC1 overexpression promotes glycolysis and epithelial-mesenchymal transition (EMT), driving cisplatin/oxaliplatin resistance.	HKDC1 knockdown restores chemosensitivity and suppresses metastasis.	(132)
MCM10	Glycolysis-driven cancer stemness	MCM10 upregulates glycolytic enzymes to sustain paclitaxel (PTX) resistance.	MCM10 knockdown inhibits tumor sphere formation and PTX resistance.	(148)
RORα	RORα-G6PD/PFKFB3 transcriptional regulation	RORα binds to G6PD/PFKFB3 promoters to suppress glycolysis; its loss enhances glycolysis and fluorouracil resistance.	RORα agonists suppress glucose uptake and reverse metabolic-driven resistance.	(149)
SLC7A5	SLC7A5-mediated glycolysis	SLC7A5 overexpression activates glycolytic genes, sustaining oxaliplatin resistance.	SLC7A5 knockdown inhibits glycolysis and restores oxaliplatin sensitivity.	(147)
Metabolic Intervention Strategies				
GO-Y022 + 2-DG	Dual targeting of Tregs and glycolysis	GO-Y022 inhibits regulatory T cells (Tregs); 2-DG blocks lactate production.	Combined therapy synergistically suppresses Treg activity and tumor growth.	(150)
Isoliquiritigenin (ISL)	Natural flavonoid targeting GLUT4/PDHK1/PGC-1α	ISL downregulates GLUT4 to impair glucose uptake and upregulates PDHK1 to suppress OXPHOS/glycolysis.	ISL induces ROS accumulation and metabolic collapse.	(151)
CS-NO	Polysaccharide-based YAP/TAZ inhibitor	CS-NO suppresses YAP/TAZ signaling, downregulating glycolytic enzymes.	CS-NO inhibits aerobic glycolysis and metastasis.	(152)
Thioridazine	Skp2/AKT/mTOR/GLUT1 axis inhibition	Thioridazine downregulates Skp2, reducing GLUT1-mediated glucose uptake.	Thioridazine synergizes with trastuzumab/lapatinib in HER2-positive GC models.	(141)
IACS-010759 + NCI-006	Dual OXPHOS/glycolysis inhibition	OXPHOS inhibitor (IACS-010759) combined with glycolysis blocker (NCI-006) induces energy crisis.	Dual inhibition prevents metabolic flexibility and enhances tumor suppression.	(153)

### 6.1 Glycolytic drivers of 5-fluorouracil resistance

The lncRNA SNHG16 (116) orchestrates 5-FU resistance by sequestering miR-506-3p, which derepresses the RNA-binding protein PTBP1. Elevated PTBP1 stabilizes glycolytic enzyme transcripts, prolonging mRNA half-lives by 1.9-fold and amplifying extracellular acidification rates (ECAR) by 67%. Clinically, SNHG16 copy number amplification and plasma levels exceeding 5.8 copies/μL predict treatment failure. Preclinical intervention using LNA inhibitors targeting SNHG16 reduces tumor burden by 58% in PDX models, demonstrating therapeutic potential.

### 6.2 Cisplatin/oxaliplatin resistance: metabolic coupling and redox adaptation

HKDC1 drives dual metabolic adaptations (132), enhancing HK activity and mitochondrial membrane potential to couple glycolysis with OXPHOS while inducing EMT. Its overexpression correlates with peritoneal metastasis risk. Concurrently, Solute Carrier Family 7 Member 5 (SLC7A5) (147) elevates intracellular α-KG, stabilizing HIF-1α to upregulate GLUT1 and LDHA, thereby increasing oxaliplatin IC50 by 3.7-fold. SLC7A5 silencing reduces lactate production by 62% and restores apoptotic sensitivity, highlighting metabolic transporters as actionable targets.

### 6.3 Paclitaxel resistance: stemness and mitochondrial reprogramming

Minichromosome Maintenance Complex Component 10 (MCM10) (148), overexpressed 4.2-fold in resistant tumors, activates Aldehyde Dehydrogenase 1 Family Member A1(ALDH1A1) and SRY-Box Transcription Factor 2 (SOX2) to promote stemness, evidenced by tumor sphere enlargement. Mechanistically, MCM10 facilitates HK2 mitochondrial translocation, suppressing Bax-mediated apoptosis. Nanoparticle-delivered MCM10 siRNA reduces cancer stem cell populations from 23% to 6% when combined with paclitaxel, offering a dual-targeting strategy.

### 6.4 Fluorouracil resistance: retinoic acid receptor-related orphan receptor alpha loss and metabolic-reparative crosstalk

Nuclear receptor RORα (149) suppresses the pentose phosphate pathway by binding G6PD and PFKFB3 promoters, reducing NADPH/NADP^+^ ratios. RORα deficiency correlates with elevated PET-CT SUVmax and circulating tumor cells, predicting resistance. Restoring RORα expression enhances 5-FU sensitivity 2.6-fold and disrupts glycolysis-DNA repair synergy, positioning RORα agonists as chemosensitizers.

### 6.5 Metabolic-immune synergy: targeting immunosuppressive niches

The HDAC inhibitor GO-Y022 reduces Treg differentiation by 63% via TGF-β/Smad3 inhibition. However, tumor-derived lactate via GPR81 activation counteracts this effect. Combining GO-Y022 with the glycolysis inhibitor 2-DG decreases lactate production and Treg infiltration, achieving 82% tumor regression in HER2-negative PDX models while enhancing CD8^+^ T-cell infiltration (150).

### 6.6 Multimodal metabolic suppression: energy crisis induction

Isoliquiritigenin disrupts energy homeostasis by reducing GLUT4 membrane localization (151), decreasing glucose uptake by 78%, and inhibiting PDHK1 to block pyruvate-to-acetyl-CoA conversion while suppressing OXPHOS, depleting ATP to 23% of baseline. Single-cell flux analysis reveals ISL reduces ALDH^+^ cancer stem cells from 14% to 3%, and combined with oxaliplatin, elevates objective response rates (ORR) to 68% in preclinical trials.

### 6.7 Nanodelivery systems: precision targeting of metabolic hubs

Chitosan-nitric oxide nanoparticles (CS-NO) exploit the enhanced permeability and retention (EPR) effect for tumor-selective accumulation (152). Sustained NO release degrades HK2 and LDHA via ubiquitination while inhibiting YAP/TAZ nuclear translocation, suppressing GLUT1 transcription. In peritoneal metastasis models, weekly CS-NO administration reduces metastatic nodules from 28 to 4, with hyperpolarized MRSI confirming an 89% lactate reduction.

### 6.8 Drug repurposing: thioridazine disrupts metabolic-growth crosstalk

Thioridazine reverses HER2^+^ GC resistance by suppressing Skp2 transcription and promoting Skp2 ubiquitination (141). This dual action reduces GLUT1 membrane localization, synergizing with trastuzumab to elevate PDX complete response rates from 22% to 59%. Phase II trials demonstrate extended median progression-free survival (mPFS) to 9.3 months versus 5.1 months with monotherapy.

### 6.9 Pan-metabolic inhibition: dual targeting of glycolysis and OXPHOS

Co-inhibition of OXPHOS and glycolysis depletes ATP to 5% of baseline, inducing RIP1/RIP3-mediated necroptosis. Hyperpolarized 13C-MRSI reveals a 92% reduction in pyruvate-to-lactate conversion, correlating with treatment response. This regimen achieves a 73% disease control rate (DCR) in metastatic GC, with metabolic imaging enabling efficacy assessment within 48 hours (153).

## 7 Emerging therapeutic targets and regulatory mechanisms

Emerging therapeutic paradigms in GC are uncovering novel regulatory layers spanning microbial ecosystems, nuclear metabolic compartmentalization, and stress-responsive redox circuits. Microbiota-metabolite interactions reshape chemosensitivity through metabolite-driven transcriptional repression of glycolytic effectors, while transcriptional-epigenetic networks enforce metabolic addiction via chromatin remodeling and Ca^2+^ signaling activation. Spatial reorganization of nuclear enzyme complexes under therapy stress creates drug-resistant niches, and redox adapters modulate nutrient deprivation responses through autophagy-microtubule dynamics. These mechanisms are exploitable via engineered probiotics, lactylation-targeted epigenetic editing, nuclear HSP90 inhibitors, and redox disruptors. The molecular architecture and therapeutic vulnerabilities of these emerging targets, alongside their clinical translation potential, are comprehensively detailed in **Table 5**, providing a roadmap for next-generation interventions against GC’s adaptive survival programs.

**Table 5:** Novel regulators and therapeutic candidates in gastric cancer.

Regulator	Target	Mechanism	Key Findings	Reference
Microbial and Metabolic Interventions				
Akkermansia muciniphila & PEA	Gut microbiota-derived PEA	PEA suppresses FUBP1 to inhibit glycolysis, enhancing oxaliplatin (OXA) efficacy.	PEA restores OXA sensitivity in dysbiotic GC models.	(154)
Transcriptional and Epigenetic Targets				
BHLHE40/GRIN2D axis	BHLHE40	BHLHE40 activates GRIN2D transcription, enhancing glycolysis and malignant progression.	GRIN2D knockdown suppresses GC growth and metabolic activity.	(155)
H3K18la/SIRT1 feedback loop	SIRT1	SIRT1 deficiency elevates H3K18la levels, upregulating lncRNA H19 and glycolysis.	SIRT1 loss correlates with poor prognosis and H3K18la accumulation.	(128)
Enzyme Complexes and Nuclear Localization				
HSP90-mediated HGEO complex	HSP90	HSP90 assembles nuclear glycolytic enzymes (PGK1/PKM2/ENO1/LDHA) to promote chemoresistance.	High HGScore predicts aggressive phenotypes and treatment resistance.	(156)
Redox and Stress Response				
ETHE1	ETHE1	ETHE1 sustains aerobic glycolysis by maintaining redox balance and ATP production.	ETHE1 knockdown reduces tumor growth and induces apoptosis.	(157)
MZT1	MZT1	MZT1 stabilizes NEDD1 to enhance glycolysis under glucose deprivation.	MZT1 overexpression promotes chemoresistance and metastasis.	(158)

### 7.1 Microbiota-metabolite synergy in chemosensitization

The gut microbiota emerges as a critical modulator of chemotherapeutic response in GC, with Akkermansia muciniphila and its metabolite pentadecanoic acid (PEA) demonstrating potent metabolic intervention capabilities (154). Antibiotic-induced dysbiosis reduces A. muciniphila abundance by 4.1-fold, diminishing oxaliplatin’s antitumor efficacy through upregulation of glycolytic enzymes. Fecal microbiota transplantation restores oxaliplatin sensitivity by downregulating LDHA and HK2 expression, while metabolomic profiling identifies PEA as a key effector—its direct binding to far-upstream element-binding protein 1 (FUBP1) suppresses HK2/GLUT1 transcription and enhances DNA damage. Clinically, elevated fecal A. muciniphila levels correlate with improved ORR. Engineered A. muciniphila delivering PEA achieves 89% complete response rates in orthotopic GC models, with oral probiotic formulations extending mPFS to 9.1 months versus 5.4 months in controls.

### 7.2 Transcriptional-epigenetic crosstalk in metabolic addiction

The basic helix-loop-helix family member BHLHE40 drives glycolytic dependency by binding the GRIN2D promoter, upregulating the NMDA receptor subunit to activate Ca^2+^/CaMKII signaling (155). This cascade enhances HK2 and LDHA expression, elevating extracellular acidification and correlating with PET-CT SUVmax. BHLHE40-high patients exhibit reduced 3-year survival and accelerated peritoneal dissemination. Concurrently, histone H3K18 lactylation—elevated 3.2-fold in metastatic GC—activates lncRNA H19 by recruiting bromodomain protein BRD4, which stabilizes PKM2 and suppresses SIRT1 (128). Therapeutic co-targeting using SIRT1 activators and LDHA inhibitors reduces lactylation while elevating apoptosis. Emerging strategies include nanoparticle-delivered BHLHE40 siRNA and lactylation-driven prognostic scoring systems.

### 7.3 Spatial metabolic organization: nuclear enzyme complexes

The HSP90-organized HGEO complex (156) coordinates nuclear translocation of glycolytic enzymes, forming metabolically active assemblies that enhance lactate production and NAD^+^/NADH ratios. Under cisplatin stress, nuclear HGEO aggregation promotes ribosome biogenesis and ATP-binding cassette (ABC) transporter upregulation, facilitating drug efflux. HSP90 inhibitors dissociate HGEO, reducing nuclear HK2 localization and restoring cisplatin sensitivity. Clinically, a HSP90 nuclear localization score predicts poor outcomes, with brain-penetrant HSP90 inhibitors extending survival in leptomeningeal metastasis models by 58 days versus controls.

### 7.4 Redox homeostasis and nutrient stress adaptation

ETHE1 overexpression stabilizes mitochondrial sulfide metabolism, maintaining redox balance and conferring cisplatin/radiation resistance (157). Conversely, glucose deprivation induces microtubule-associated protein MZT1 to stabilize EB1 and CLASP2 (158), activating ULK1-mediated autophagy and promoting tumor survival. MZT1-high patients exhibit 3.1-fold higher recurrence risk and reduced bevacizumab response. Pharmacological inhibition of ETHE1 or MZT1 disrupts redox adaptation and microtubule dynamics, enhancing chemoradiation efficacy while suppressing metastatic outgrowth.

## 8 Discussion and conclusion

The systematic dissection of metabolic reprogramming in GC has unveiled intricate molecular networks that sustain tumor progression and therapeutic resistance. Mechanistic advances delineating the crosstalk between oncogenic signaling, epigenetic remodeling, and microenvironmental adaptation have positioned metabolic plasticity as a linchpin of malignant survival (159–163). Key regulatory nodes within glycolytic flux, redox balancing, and stress response pathways—including HIF-α/AMPK/mTOR signaling, RNA-modified enzyme complexes, and microbiota-metabolite interactions—now offer actionable targets for therapeutic exploitation (121, 145, 164–167). Emerging strategies extend beyond enzymatic inhibition to encompass microbial modulation and spatial metabolic disruption, reflecting the multidimensional nature of cancer metabolism (168–170). Despite significant advancements in GC research, several critical barriers remain in translating preclinical findings to clinical applications. One major challenge is the inability of current preclinical models to replicate the metabolic heterogeneity observed across different GC molecular subtypes and metastatic microenvironments. GC tumors undergo extensive metabolic reprogramming, yet preclinical models often fail to reflect the complexity of these processes, which vary depending on factors such as genetic mutations and the tumor microenvironment. Additionally, the dynamic interplay between metabolic changes and immune evasion, particularly in therapy-resistant niches enriched with immunosuppressive cells like regulatory T cells (Tregs) and M2 macrophages, is still not fully understood. The lack of standardized metabolic profiling protocols further complicates the comparison of results across studies, limiting the clinical applicability of findings. Addressing these challenges requires the development of standardized techniques for metabolic profiling, which could enable the identification of patients most likely to benefit from targeted therapies.

To overcome these hurdles, a multifaceted approach is needed, involving clinical innovation, technological advancements, and a deeper mechanistic understanding of tumor biology. Clinical trials, particularly those in phase II/III, should consider integrating metabolic inhibitors—such as LDHA blockers or SIRT1 activators—with chemotherapy or immunotherapy. These studies could benefit from biomarker-enriched cohorts, utilizing advanced tools like hyperpolarized ¹³C-MRI or ctDNA-based metabolic signatures for real-time monitoring of therapeutic efficacy. Furthermore, emerging technologies such as spatial metabolomics and single-cell flux analysis could provide detailed insights into the metabolic dependencies of GC subtypes, facilitating the identification of subtype-specific vulnerabilities for targeted therapy. AI-driven multi-omics integration could also help predict adaptive resistance mechanisms, such as the reactivation of OXPHOS in response to therapy, offering new avenues for overcoming resistance. Mechanistically, future research should focus on how tumor-derived metabolites—such as lactate and ketone bodies—interact with immune cells to modulate immune responses, particularly in relation to T cell exhaustion and immune evasion. For example, succinate accumulation in SDH-deficient GC may activate SUCNR1 on dendritic cells, suppressing antitumor immunity. Although still in preclinical development, SUCNR1 antagonists are being explored as potential therapeutic agents to reverse this immune suppression. Additionally, strategies such as nanoparticle-mediated delivery of HK2 inhibitors to TAMs could help reverse their immunosuppressive polarization, enhancing systemic immunity while targeting the tumor microenvironment. These integrated approaches, combining metabolic reprogramming with immune modulation, offer promising directions for developing more effective and personalized therapies for GC in the future.

PKM2, a glycolytic enzyme, has been found to be highly expressed in many cancers, including GC. In the context of exosomes, PKM2 - loaded exosomes released by cancer cells can be taken up by neighboring cells, such as macrophages in the tumor microenvironment. Macrophages, which are integral components of the immune system, can be polarized into different phenotypes in the tumor setting. Tumor - associated macrophages (TAMs) often exhibit an M2-like phenotype that promotes tumor growth, invasion, and metastasis. Emerging data suggest that exosomal PKM2 plays a role in activating lipid synthesis pathways in macrophages. Once macrophages internalize exosomal PKM2, it can modulate intracellular signaling pathways. PKM2 may act as a protein kinase in addition to its enzymatic function. It can phosphorylate key regulators involved in lipid metabolism. For example, it might phosphorylate transcription factors that are crucial for the activation of genes encoding enzymes in the lipid synthesis pathway, such as acetyl - CoA carboxylase (ACC) and fatty acid synthase (FAS). Activation of these enzymes leads to increased *de novo* lipid synthesis in macrophages (171).

Increased lipid synthesis in macrophages has several implications for GC progression. Lipids can serve as building blocks for cell membranes, which is important for the rapid proliferation of cancer cells. Macrophages with enhanced lipid synthesis can secrete lipid - rich vesicles or directly transfer lipids to cancer cells, providing them with the necessary resources for growth and division. Moreover, lipids can also function as signaling molecules. For instance, certain lipid species can activate signaling pathways in cancer cells that promote their migration and invasion, processes that are critical for GC metastasis. Furthermore, the activation of lipid synthesis pathways in macrophages by exosomal PKM2 may also contribute to the immunosuppressive tumor microenvironment. M2 - like macrophages are known to secrete cytokines and chemokines that can inhibit the function of immune effector cells, such as T - lymphocytes. By enhancing lipid synthesis, exosomal PKM2 may further skew macrophages towards an immunosuppressive phenotype, thus allowing cancer cells to evade immune surveillance (172). Looking ahead, emerging technologies offer promising avenues to target the exosomal PKM2-lipid synthesis axis in gastric cancer. AI-based metabolic imaging, for instance, could enable non-invasive visualization of lipid metabolic reprogramming in tumor-associated macrophages (TAMs) within the gastric cancer microenvironment. By integrating high-resolution imaging data with machine learning algorithms, this approach might precisely map spatiotemporal changes in lipid synthesis triggered by exosomal PKM2, facilitating early detection of microenvironmental dysfunction and personalized treatment monitoring. Meanwhile, CRISPR-based metabolic reprogramming presents another frontier. Engineered CRISPR systems could be tailored to disrupt PKM2 expression or its downstream signaling in macrophages, directly inhibiting exosome-induced lipid synthesis pathways. Such strategies might selectively reverse the pro-tumorigenic phenotype of TAMs without disrupting systemic lipid metabolism, offering a novel precision therapy to normalize the tumor microenvironment and enhance responsiveness to existing treatments.

Overall, glycolytic reprogramming is a hallmark of GC progression, driving therapy resistance and metabolic adaptability. Key regulatory nodes in glycolysis—from HK-mediated glucose trapping to lactate-fueled microenvironment remodeling—offer promising therapeutic targets. While preclinical advances in targeting glycolytic enzymes and signaling hubs demonstrate efficacy, clinical translation requires overcoming tumor heterogeneity and adaptive resistance mechanisms. Future progress hinges on integrating glycolytic biomarkers, advanced metabolic imaging, and innovative delivery systems to enable precision targeting of this central metabolic vulnerability, ultimately reshaping therapeutic paradigms for GC.

## Glossary

GC: Gastric cancer
TME: tumor microenvironment
HK2: hexokinase 2
LDHA: lactate dehydrogenase A
HIF-1α: hypoxia-inducible factor-1α
GLUT: glucose transporter
HK: hexokinase
PFK-1: phosphofructokinase-1
G6P: glucose-6-phosphate
F6P: fructose-6-phosphate
F1,6BP: fructose-1,6-bisphosphate
F2,6BP: fructose-2,6-bisphosphate
GA3P: glyceraldehyde-3-phosphate
LDH: lactate dehydrogenase
OXPHOS: oxidative phosphorylation
ETC: electron transport chain
L-2-HG: L-2-hydroxyglutarate
α-KG: α-ketoglutarate
VDACs: voltage-dependent anion channels
EMT: epithelial-mesenchymal transition
CTL: cytotoxic T lymphocyte
TAMs: tumor-associated macrophages
PKM2: Pyruvate kinase M2
circRNA: circular RNA
ceRNA: competitive endogenous RNA
PGK1: phosphoglycerate kinase 1
RRM2: ribonucleotide reductase M2
lncRNAs: Long non-coding RNAs
EBV: Epstein-Barr virus
EBVaGC: EBV-associated GC
PTMs: post-translational modifications
MCCC2: methylcrotonoyl-CoA carboxylase 2
PROTACs: proteolysis-targeting chimeras
LNA: locked nucleic acid
ARRB1: β-arrestin 1
ALDOB: aldolase B
18F-FLT: 18F-fluorothymidine
PDX: patient-derived xenograft
LPA: Lysophosphatidic acid
Gyp: Ginkgolide
ECAR: extracellular acidification rates
ORR: objective response rates
CS-NO: Chitosan-nitric oxide nanoparticles
mPFS: median progression-free survival
DCR: disease control rate
PEA: pentadecanoic acid
FUBP1: far-upstream element-binding protein 1
ABC: ATP-binding cassette
Tregs: regulatory T cells
HER2: Human epidermal growth factor receptor 2
TNM: Tumor node metastasis classification
EZH2: enhancer of zeste homolog 2
PTBP1: polypyrimidine tract-binding protein 1
LATS2: Large Tumor Suppressor Kinase 2
TGFB1: Transforming Growth Factor Beta 1
DLEU1: Deleted in Leukemia 1
APOC1: Apolipoprotein C1
SNP: Single Nucleotide Polymorphism
c-MYC: cellular myelocytomatosis oncogene
PDLIM1: PDZ and LIM Domain Protein 1
DDX24: DEAD-box Helicase 24
HKDC1: Hexokinase Domain Containing 1
SHP2: Src Homology 2 Domain-Containing Protein Tyrosine Phosphatase 2
18F-FDG-PET: Fluorine-18 Fluorodeoxyglucose Positron Emission Tomography
STAT3: Signal Transducer and Activator of Transcription 3
PGAM1: Phosphoglycerate mutase 1
TOP1MT: Topoisomerase 1 Mitochondrial
PDK4: Pyruvate Dehydrogenase Kinase 4
METTL14: Methyl transferase-like 14
CCL7: C Motif Chemokine Ligand 7
SLC7A5: Solute Carrier Family 7 Member 5
MCM10: Minichromosome Maintenance Complex Component 10
ALDH1A1: Aldehyde Dehydrogenase 1 Family Member A1
SOX2: SRY-Box Transcription Factor 2
RORα: Retinoic Acid Receptor-Related Orphan Receptor Alpha
EPR: enhanced permeability and retention
GLUT1: Glucose transporter 1
SNHG16: Small Nucleolar RNA Host Gene 16
uMtCK: ubiquitous mitochondrial creatine kinase
